# Cloning of a synthetic chimeric gene containing recombinant *Mycoplasma hyopneumoniae *antigens for expression in *Pichia pastoris*

**DOI:** 10.1186/1753-6561-8-S4-P247

**Published:** 2014-10-01

**Authors:** Charles Gomes, Andressa Fisch, Silvana Marchioro, Lucas Tavares, Natasha Oliveira, Ana Peiter, Sérgio Jorge, Odir Dellagostin

**Affiliations:** 1 Laboratório de Biologia Molecular, Núcleo de Biotecnologia, Centro de Desenvolvimento Tecnológico, Universidade Federal de Pelotas, Pelotas, Brazil

## Background

*Mycoplasma hyopneumoniae *is the primary etiologic agent of Swine Enzootic Pneumonia (EP), one of the most common respiratory disease that affects swine worldwide, causing considerable economic losses. Vaccination constitute one of the main practices to control EP. The production of recombinant experimental vaccines against EP has been considered an important approach towards the development of an improved vaccine [1]. This study aimed to clone a synthetic gene composed by the fusion of *M. hyopneumoniae *antigens R1 (P97), P42 and NrdF to *Escherichia coli *fragment B of the heat labile enterotoxin (LTB) into the vectors pPICZB (intracellular expression) and pPICZαB (secreted expression), in order to produce this chimeric recombinant protein in *Pichia pastoris *and to test it as a vaccine candidate. The *M. hyopneumoniae *antigens were selected due to their capacity to confer partial protection in pigs when evaluated individually [2-4]. These antigens were associated to LTB, a potent mucosal and parenteral adjuvant.

## Methods

The DNA fragment coding for the recombinant chimeric protein was designed in silico [5]. The synthetic gene and the vectors pPICZαB and pPICZB were digested with the enzymes *Bam*HI and *Eco*RI and ligated. The ligation product was transformed into *E. coli *TOP10 strain, plated on Luria - Bertani (LB) culture medium containing the antibiotic Zeocin™ (Invitrogen). The resulting colonies were selected through a rapid screening method using a protocol with phenol - chloroform. Recombinant clones were expanded in LB liquid medium with antibiotic, the plasmid was extracted and then characterized with restriction enzymes *Bam*HI and *Eco*RI.

## Results and conclusions

The synthetic gene coding for the chimeric protein, composed by the fusion of *M. hyopneumoniae *antigens R1 (P97), P42, NrdF and to LTB were efficiently cloned into the two expression vectors in *P. pastoris*, pPICZB and pPICZαB. Digestion of the recombinant clones resulted in the release of a fragment of 1500 pb, corresponding to the size of the insert. Future tests will be performed with these clones in order to optimize expression of this chimeric protein in *P. pastoris*.
